# Conventional versus Monte Carlo SPECT reconstruction of Lu‐177: Toward reduced bias and variance in quantitative imaging

**DOI:** 10.1002/mp.70222

**Published:** 2025-12-21

**Authors:** Lucas A. Polson, Pedro Esquinas, Sara Kurkowska, Chenguang Li, Carlos Uribe, Arman Rahmim

**Affiliations:** ^1^ Department of Physics & Astronomy University of British Columbia Vancouver Canada; ^2^ Department of Integrative Oncology BC Cancer Research Institute Vancouver Canada; ^3^ Molecular Imaging and Therapy Department BC Cancer Research Institute Vancouver Canada; ^4^ Department of Nuclear Medicine Pomeranian Medical University Szczecin Poland; ^5^ Department of Radiology University of British Columbia Vancouver Canada

**Keywords:** Monte Carlo, reconstruction, spect

## Abstract

**Background:**

Monte Carlo (MC)‐based single photon emission computed tomography (SPECT) reconstruction utilizes advanced system models that stochastically sample all possible photon interactions within the patient and detector, potentially increasing quantitative accuracy and precision. Despite this, there have been few studies that have rigorously compared conventional SPECT reconstruction and MC‐based reconstruction for metrics that are pertinent to radiopharmaceutical dosimetry.

**Purpose:**

This paper aims to compare conventional reconstruction with hybrid‐MC reconstruction and explores accuracy and precision in total activity estimation within various regions of interest in 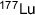 imaging.

**Methods:**

The MC engine Simulation of Medical Imaging Nuclear Detectors (SIMIND) was integrated with the reconstruction software PyTomography to enable MC‐based reconstruction. The following are explored: (i) elimination of triple energy window (TEW)‐induced bias obtained by using an MC system model and (ii) improvements to precision and effective reductions in required scan time that are attained when using MC‐based reconstruction. The study explores multiple acquisitions of simulated and real phantom/patient data.

**Results:**

Conventional reconstruction with TEW induces a positive bias (e.g., 116.3% recovery coefficient, or RC, in a 72 mm sphere from the MC‐simulated data) that is not present with MC‐based reconstruction (e.g., 101.3 RC%). This source of positive bias raises RC in smaller spheres, effectively canceling with negative bias incurred from finite resolution; in real phantom and patient data, RCs are lower with MC‐based reconstruction than conventional, suggesting that MC‐based reconstruction is able to remove the additional source of bias caused by TEW. Furthermore, MC‐based reconstruction is able to reduce variability between subsequent acquisitions of the same phantom (e.g., reducing variability by (4.19±1.39)% in 10 mm sphere), thus resulting in reconstructed images comparable to those obtained with longer scan times (e.g., by a factor of 2.78±0.33 in the 32 mm sphere).

**Conclusions:**

Compared to conventional reconstruction that uses TEW to correct for scatter, MC‐based reconstruction for 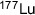 (i) reduces sources of systematic bias caused by inadequate TEW scatter estimation and (ii) reduced required scan times by reducing total uptake variability in regions of interest across different scans. For this reason, MC‐based reconstruction should be preferred to conventional reconstruction in clinical practice.

## 1 INTRODUCTION

Radiopharmaceutical therapies (RPTs) are a rapidly growing modality in cancer treatment, with FDA and EMA approved applications of 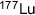 for treatment of neuroendocrine tumors^1^, ^2^, ^3^, ^4^, ^5^ and prostate cancer;^6^, ^7^, ^8^, ^9^ these approved drugs consist of a combination of targeting molecules that yield increased biodistribution near pathological tissue, and a radioactive component that delivers localized damage via $\beta^{-}$ emissions. Since 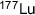
‐based radiopharmaceuticals also emit γ particles, they can be imaged using a single photon emission computed tomography (SPECT) camera. It is postulated that monitoring of patient radiation absorbed dose via multi‐time point quantitative SPECT imaging post‐treatment can be used to tailor patient‐specific activity injections and improve therapeutic outcome, for example, by prescribing larger injected activities in later cycles to patients that had lower uptake in earlier cycles.^10^ There has been a recent interest on how healthy organ dosimetry can predict survival and toxicities in 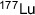
‐DOTATATE treatments;^11^ others have explored absorbed dose thresholds for partial response in grade 2 neuroendocrine tumors.^12^ Since dosimetry relies on SPECT imaging, it is necessary to consider error in activity quantification, which is comprised of both accuracy and precision.

Degradation of accuracy in SPECT images may result from the following: (i) an inaccurate system model used in image reconstruction or (ii) finite resolution of an imaging system:

1. In this work, it will be demonstrated that the patient scatter estimates obtained using the triple energy window (TEW) method,^13^ which estimates the scattered pNhotons through projections acquired in adjacent energy windows to the photopeak, yield inaccuracies in the 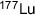 imaging. The theoretical explanation is that TEW relies mostly on photons from lower adjacent energy windows to the photopeak; these lower windows contain photons that have scattered on average by larger angles, effectively causing a blurred estimate of scatter projection and underestimating scatter counts in hot regions (see Appendix A). This consequently leads to overestimation of hot regions relative to warm regions after reconstruction (i.e., positive bias in contrast).
2. Finite resolution in an imaging system, is a hardware‐related limitation that causes blurring, and while loss of resolution can be included in system models,^14^, ^15^ it cannot be fully reversed during image reconstruction, since finite resolution permits nonunique solutions. Blurring corresponds to an underestimation of activity concentration in sufficiently small region of interests (ROIs), yielding inaccuracies in reconstructed images.

Monte Carlo (MC)‐based reconstruction approaches^16^, ^17^ simulate billions of individual photons to model all relevant physical phenomena in an imaging system including patient attenuation/scatter as well as the response in the SPECT system. Importantly, while MC‐based reconstruction approaches can be used to improve system models (point i), they fundamentally cannot overcome limited resolution (point ii).

Degradation of precision, on the other hand, is due to the statistical nature of the counts acquired in a SPECT scan; longer scans and higher activities yield improved precision owing to more counts acquired. Our recent work^18^ explored precision in emission tomography, expanding on prior work of Qi^19^ to develop an algorithm that estimates precision of ROI total activity in reconstructed images based on uncertainty in the raw projection data. The conventional reconstruction approaches explored used both a photopeak window as well as two adjacent scatter windows to correct for scatter via TEW; this is in contrast to a MC‐based reconstruction approach,^16^, ^17^ which models scatter implicitly in the system matrix. The reliance on additional noisy scatter window data in conventional reconstruction will be demonstrated to worsen the precision in reconstructed images. In this paper, precision is quantified as total uptake variability across different noise realizations (e.g., via multiple scans of an identical phantom).

Image reconstruction algorithms utilize a system model (which can be represented as a matrix) that models the necessary physics of the imaging system (see Section 2.1). The MC‐based reconstruction technique in this paper is denoted “hybrid” because it uses an MC‐derived system model for forward matrix multiplication, but uses analytical system modeling (that does not consider patient‐scatter) when the transpose operation is used. This terminology is intentionally used to differentiate from “full” MC reconstruction that uses MC in both operators, though it should be noted such a technique has not yet been developed or studied for SPECT. Hybrid‐MC reconstruction has previously been used by Gustafsson^17^ and Rydén^16^ in 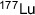 image reconstruction, though a rigorous analysis of bias and variance in reconstructed images for purposes of dosimetry has not yet been performed. The purpose of this paper is to compare the total bias and variability in ROI mean values in 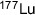 images obtained with a conventional versus MC‐based SPECT reconstruction approach. These metrics are particularly important for organ and lesion level dosimetry, which relies on total activity quantification in regions consisting of multiple voxels. The reconstruction software used here is Pytomography^20^ integrated with Simulation of Medical Imaging Nuclear Detectors (SIMIND)^21^ as an MC engine; the use of MC‐based reconstruction is made publicly available.

## 2 MATERIALS AND METHODS

### 2.1 Theory

The maximum likelihood expectation maximization (MLEM) algorithm corresponds to the ordered subset expectation maximization (OSEM)^22^ with one subset, and can be expressed as

(1) $${\hat{f}}^{\left(k+1\right)}={\hat{f}}^{\left(k\right)}\frac{H^{T}\left(\frac{g}{Hf+\hat{\bar{a}}}\right)}{H^{T}1},$$

where ${\hat{f}}^{k}$ is the image estimate after k iterations, g is the raw image (projection) data, H is a system matrix that models a partial response from the acquired signal, and $\hat{\bar{a}}$ is an additive term estimate that accounts for all other photons in the acquired photopeak window that are not modeled by the system matrix. The iterative algorithm is looped over to update an initial image estimate ${\hat{f}}^{\left(0\right)}$ and guarantees convergence with increasing iterations. There are two cases considered in this paper:

- **Conventional reconstruction**: In conventional (or “analytic”) reconstruction, the system model H is comprised of mathematical operations that model photons of a single energy that do not scatter within the patient. $\hat{\bar{a}}$ must therefore account for patient scatter as well as other phenomena in the primary window, such as detector backscatter from high energy photons. For 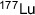, it is approximately estimated using TEW:
(2) $${\hat{\bar{a}}}_{\mathrm{TEW}}=\left(\frac{g_{\mathrm{upper}}}{w_{\mathrm{upper}}}+\frac{g_{\mathrm{lower}}}{w_{\mathrm{lower}}}\right)\cdot \frac{w_{\mathrm{peak}}}{2}$$
- **MC‐based reconstruction**: In MC‐based reconstruction, H in the forward operation Hf is replaced by an MC simulation in which billions of photons are individually simulated to model all relevant phenomena in the imaging scenario, including patient scatter, collimator penetration/scatter, and detector backscatter. In this case, the additive term only needs to account for stray background radiation (which is particular to a given imaging environment); this contribution is ignored in this work because the number of acquired background counts is negligible relative to the counts from 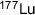.

It follows that conventional reconstruction relies on an additive term estimate ${\hat{\bar{a}}}_{\mathrm{TEW}}$ that depends on noisy data, which MC‐based reconstruction does not need; it will be demonstrated that this yields inferior accuracy and precision.

### 2.2 Metrics

The metrics used in this paper consider (i) accuracy and (ii) precision, and are represented by biases and variability in total ROI uptake, respectively. The estimated total counts $\hat{C}$ in an ROI ξ is given by

(3) $$\hat{C}\left(\xi \right)=w{\hat{f}}^{k}\cdot \xi ,$$

where “·” is the dot product, ξ is a 3D image mask consisting of either ones or zeros, and w is a scalar weighting factor that accounts for activity decay when comparing different acquisitions from real data. In a controlled phantom scan, a background ROI $\xi_{\mathrm{bkg}}$ can be used to derive a calibration factor, which converts counts to activity, as follows:

(4) $$\mathrm{CF}=\frac{A(\xi_{\mathrm{bkg}})}{\hat{C}\left(\xi_{\mathrm{bkg}}\right)},$$

where $A(\xi_{\mathrm{bkg}})$ is the known background activity in the ROI and $\hat{C}\left(\xi_{\mathrm{bkg}}\right)$ is the estimated counts in the ROI. This technique assumes that the total counts in an ROI placed in a homogeneous background do not suffer resolution degradation. The RC in an arbitrary ROI ξ can then be computed as

(5) $$\mathrm{RC}\left(\xi \right)=\frac{\hat{C}\left(\xi \right)\cdot \text{CF}}{A(\xi )}=\frac{\hat{C}\left(\xi \right)/\hat{C}\left(\xi_{\mathrm{bkg}}\right)}{A\left(\xi \right)/A\left(\xi_{\mathrm{bkg}}\right)}.$$

Equation (5) corresponds to a single noise realization; when RC is computed from averaging N noise realizations, the standard error can be computed as the standard deviation divided by $\sqrt{N}$ (provided all N realizations are independent, identically distributed random variables). The precision of the counts within an ROI σ (across N measurements) in a reconstructed image is quantified by uptake variability between scans, defined as

(6) $$\sigma \left(\xi \right)=\frac{\sqrt{\mathrm{Var}\left[\left\{{\hat{C}}_{i}\left(\xi \right)\;;\;1\leq i\leq N\right\}\right]}}{\mathrm{Mean}(\left[\left\{{\hat{C}}_{i}\left(\xi \right)\;;\;1\leq i\leq N\right\}\right])}.$$

The variability is thus expressed in units of percent standard deviation. The argument ξ will henceforth be assumed implicit. The first comparative metric for variability considered in this work is the difference

(7) $$\Delta \equiv \sigma_{\mathrm{conv}}-\sigma_{\mathrm{MC}}.$$

When this difference is positive, the variability in MC reconstruction is smaller, demonstrating advantages in using MC‐based reconstruction. We show (see appendix) that the uncertainty in Δ itself can be estimated via

(8) $$\hat{u}\left[\Delta \right]\approx \frac{1}{\sqrt{2(N-1)}}\sqrt{\sigma_{\mathrm{conv}}^{2}+\sigma_{\mathrm{MC}}^{2}-\frac{2\;\hat{\mathrm{Cov}}{\left({\hat{C}}_{\mathrm{conv}},{\hat{C}}_{\mathrm{MC}}\right)}^{2}}{\sigma_{\mathrm{conv}}\sigma_{\mathrm{MC}}}},$$

where Cov is the covariance between the regional counts ${\hat{C}}_{\mathrm{conv}}$ and ${\hat{C}}_{\mathrm{MC}}$, accross different noise realizations for each reconstruction technique; inclusion of the covariance consequently lowers the uncertainty.

We previously demonstrated (Figure 10 in Polson et al.^18^) that variability in total activity, within a fixed ROI and across separate noise realizations, is proportional to the square root of total reconstructed counts within the ROI. Since total counts are linearly proportional to acquisition time, the following relationship holds:

(9) $$\frac{t_{2}}{t_{1}}={\left(\frac{\sigma_{2}}{\sigma_{1}}\right)}^{2},$$

where $t_{i}$ is the total scan time and $\sigma_{i}$ is the count variability in some ROI. If the variability $\sigma_{1}$ for a given scan acquired for time $t_{1}$ is known, then to get a variability of $\sigma_{2}$ (for an identical ROI in an identical patient), the scan must be acquired for a time $t_{2}$. As such, the present work considers the following metric, denoted the “speed‐up factor”

(10) $$\mathrm{SF}\equiv {\left(\frac{\sigma_{\mathrm{conv}}}{\sigma_{\mathrm{MC}}}\right)}^{2}.$$

The SF for a given ROI denotes the relative amount of time, a conventional reconstruction would need to be acquired for to attain the same uptake variability as an MC‐based reconstruction; for example, if data was acquired for $t_{1}\;=$ 15 s/projection and the SF=3, this implies that conventional reconstruction would have required data to be acquired for 45 s/projection to reach the same variability as MC‐based. Conversely, 1/SF corresponds to the fraction of time that data would need to be acquired for, if hybrid‐MC reconstruction is used to get the same variability as conventional reconstruction. It can be shown (see appendix) that the following is an estimator for the uncertainty of the SF

(11) $$\hat{u}\left[\mathrm{SF}\right]=\mathrm{SF}\cdot \frac{2}{\sqrt{N-1}}\cdot \sqrt{1-\frac{\hat{\mathrm{Cov}}{\left({\hat{C}}_{\mathrm{conv}},{\hat{C}}_{\mathrm{MC}}\right)}^{2}}{\sigma_{\mathrm{conv}}\sigma_{\mathrm{MC}}}}.$$

### 2.3 Experiments

The following three experiments were used to evaluate accuracy and precision; real data experiments rely on imaging an identical phantom multiple times in order to compute Equation (6). In each case, data was reconstructed using (i) conventional reconstruction with TEW (as previously described mathematically in Polson et al.^20^) and (ii) a MC‐based reconstruction approach with OSEM with eight subsets and up to ten iterations. The analytical system matrix used for forward and back projection (conventional) and back projection (MC‐based) accounted for attenuation and employed source‐detector distance dependent PSF modeling.

The MC‐simulated system matrix is used to generate data in the first experiment and used in all MC reconstructions used a SIMIND model representative of a Siemens Symbia T2 system. It included a 0.9525 cm NaI crystal, a 6.6 cm Pyrex backscatter layer representative of detector electronics, and a 0.1 cm aluminum cover. SIMIND was configured to: (i) track photon interactions up to a phantom‐scatter order of 6 and detector scatter order of 10.

#### 2.3.1 Simulated spherical phantom

Acquisition of a 20 cm cylindrical phantom with three spheres (diameters 7.2, 4, and 2 cm) at a 10:1 source to background concentration was simulated using the Symbia T2 model in SIMIND. The data was collected at 96 projection angles with a 128 × 128 matrix (0.48 cm × 0.48 cm pixel size). Acquired energy windows were 187.2−228.8 keV (primary) 166.4−187.2 keV (lower), and 228.8−249.6 keV (upper). The purpose of this experiment was to explore differences in accuracy between the conventional TEW and hybrid‐TEW reconstruction approaches assuming ideal physics modeling in image reconstruction. The output of the SIMIND simulation yielded an estimate of the expected counts in each detector pixel and had negligible noise due to the large number of photons simulated. This “noiseless” output was used directly in image reconstruction in order to isolate for biases in reconstructed images that are caused purely by bias (and not noise) in the projection data. It should be emphasized that (i) the 7.2 cm sphere was used to disentangle biases between partial volume effects and inadequate scatter correction, since partial volume effects are negligible here and (ii) this represents an idealized scenario, where the MC simulation used in reconstruction perfectly encapsulates all physics in the acquired data, thus permitting study of biases inherent in the reconstruction algorithm (such as unrecoverable resolution losses) and not due to errors in physics modeling. ROI masks were obtained by taking all voxels contained entirely in the spherical region.

#### 2.3.2 NEMA phantom

A National Electronical Manufacturers Association (NEMA) phantom with spheres of diameter 37, 28, 22 , 17 , 13 , and 10 mm was filled with a 9:1 source to background ratio of 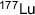 with sphere activity concentration of 0.89MBq/mL. Fifty‐three SPECT acquisitions of the phantom were taken in sequence on a Siemens Symbia T2 system with the following settings: 128×128 pixels of size 4.82mm×4.82mm, 96 projection angles, medium energy collimators, and 15 s acquisition time per projection. Acquired energy windows were identical to the previous section. Eight regions were considered to estimate RCs and uncertainty: the six spheres in the NEMA phantom, a background VOI consisting of two 50mm diameter spheres drawn in the warm region, and the central cold cylinder portion of the phantom. RC as a function of sphere radius R (at 10 iterations) were fit to a modified version of the Gustafsson–Minguez equation;^23^ the traditional equation describes the theoretical mean RCs for spherical objects convolved with a Gaussian of width w. The modified version used here introduces an additional scaling factor S that accounts for improper scatter correction:

(12) $$\begin{array}{c} \mathrm{RC}\left(R;S,w\right)=S\cdot \left[\text{erf}\left(\frac{R\sqrt{2}}{w}\right)-\frac{1}{\sqrt{2\pi }}\frac{w}{R}\right.\\ \left.\left(3-e^{-2R^{2}/w^{2}}\right)+\frac{1}{\sqrt{2\pi }}{\left(\frac{w}{R}\right)}^{3}\left(1-e^{-2R^{2}/w^{2}}\right)\right] \end{array}$$

where w is the volume overlap between two spheres of radius R a distance d apart. A value of S=1 corresponds to an ideal case, where all bias in RC values is purely due to resolution degradation. The purpose of Equation (12) is to extrapolate results of the small spheres to estimate the RC for the larger spheres.

#### 2.3.3 ‐DOTA‐TATE patient data

This subexperiment explored reconstruction of 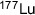
‐DOTA‐TATE RPT data from the University of Michigan Deep Blue data sharing repository.^24^, ^25^, ^26^ The time point considered was the day of injection; images were acquired on a Siemens Intevo system with a 128×128 matrix size and 120 projections with 25 s acquisition time per projection; acquired energy windows were 187.6−229.2 keV (primary) 166.7−187.6 keV (lower), and 229.2−250.1 keV (upper). To study variability across multiple acquisitions, the projection data were subsampled into six sets of projections, each consisting of 4/25 the fraction of original counts; this corresponds to acquisitions of 4 s/projection. This was accomplished by (i) randomly sampling a proportion of counts from the projection data and (ii) rebinning them to get projections with effectively less counts. Two 40 mm spheres were drawn in the liver as a background estimate; activity concentration ratios between liver lesions and liver background were computed as an analogue for RCs, as the true activity was not known. Count variability was obtained for the two lesions in the liver, the liver background, and the left/right kidneys.

## 3 RESULTS

**FIGURE 1:**
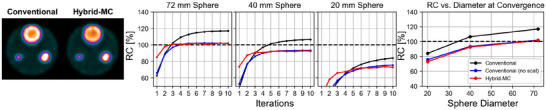
Reconstruction of SIMIND simulated cylindrical phantom containing three hot spheres in a warm background. Left: central axial slices of reconstruction, demonstrating visually higher contrast in conventional reconstruction. Right: RC curves as a function of iteration. Conventional (no scat) corresponds to SIMIND data that only consisted of primary detections.RC, recovery coefficient.

Reconstructed images and RC curves of the simulated spherical phantom are shown in Figure 1. For the 72 mm sphere, both conventional reconstruction of simulated projections without scatter (i.e., primary photons only) and MC‐based reconstruction closely converge to 100% RC (101.9% and 101.3%, respectively). When scatter events are included in the data used for conventional reconstruction, however, TEW scatter estimation causes an overestimation of contrast, resulting in a RC equal to 116.9%, indicating a positive bias associated with TEW. The overestimation of conventional versus MC‐based occurs for all sphere sizes; for the smaller 40  and 20 mm spheres, there is also a decrease in RC due to the finite resolution of the imaging system.

**FIGURE 2:**
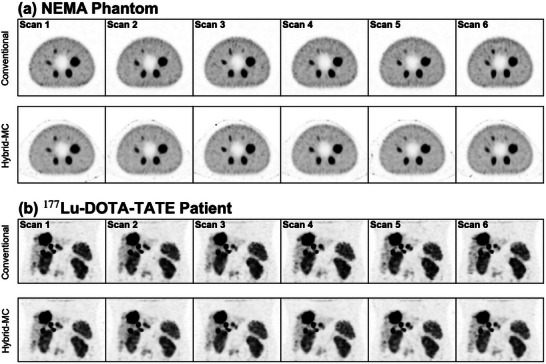
(a) Axial MIPs of reconstructed NEMA phantoms using conventional and MC‐based reconstruction; the first six noise realizations of 53 reconstructed images are shown. (b) Coronal MIPs of patient receiving 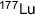
‐DOTA‐TATE therapy; separate scans correspond to six different effective 4s/projection scans that were sampled from a single scan of 25s/projection.MIPs, maximum intensity projections.

Six sample axial maximum intensity projections (MIPs) of the 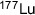 NEMA phantom are shown in Figure 2a, while six MIPs of the 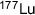
‐DOTA‐TATE patient are shown in Figure 2b. These visual results demonstrate noticeable, but minor qualitative differences between the reconstructed images: in particular, the MC‐based reconstructions appear to have less noise in the warm background (phantom) and liver (patient). This is confirmed via percent standard deviation of counts ((23.0±0.18)% vs. (19.25±0.18)% NEMA warm background and (282.7±0.6)% vs. (262.2±0.2)% patient liver).

**FIGURE 3:**
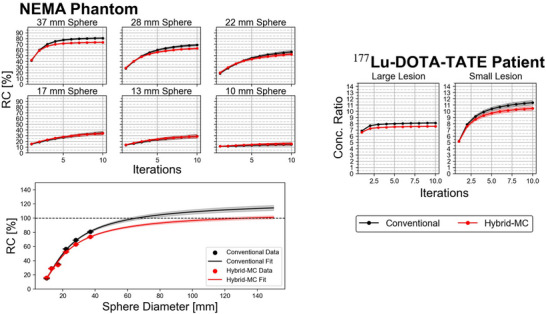
RC curves of six NEMA spheres (left) and activity concentration ratios between patient lesions and liver background (right). Error bars correspond to the standard error on the mean computed using different noise realizations. The bottom plot for the NEMA phantom shows the RC versus sphere diameter at 10 iterations, as well as an associated curve fit using Equation (12). RC, recovery coefficient.

RC curves (NEMA phantom) and concentration ratio curves (DOTA‐TATE patient) are shown in Figure 3. In each case, the slightly lower concentration ratio of MC‐based is consistent with Figure 1, suggesting that the MC‐based is better able to compensate for scatter events and consequently produce images with fewer sources of bias. For example, the percent difference in RC between conventional and MC‐based in the 32 mm sphere of −7.24% reflects an analogous difference in the 40 mm sphere of the MC‐simulated data (−14.1%). The mean lesion concentration ratio in the liver differs by −6.94% (large lesion) and −8.48% (small lesion). The fit parameters to Equation (12), were S=1.26±0.05 and w=0.57±0.03cm (conventional) versus S=1.10±0.03 and w=(0.53±0.02) cm for hybrid‐MC. This indicates (i) that hybrid‐MC may in fact have less resolution degradation (w is smaller for hybrid‐MC), and (ii) that the reason for higher RCs in conventional is purely due to bias in the scatter estimate (S is larger for conventional). Figure 3 also demonstrates clear overestimation of RCs when conventional reconstruction is used for large volumes.

**FIGURE 4:**
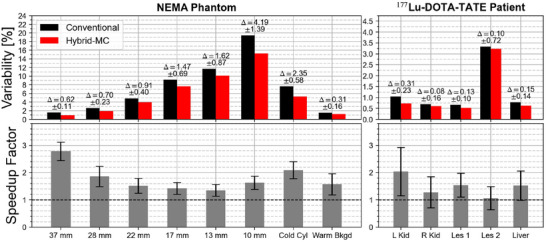
Variability and speedup factor for various ROIs in NEMA phantom (left) and 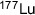
‐DOTA‐TATE patient (right). Error bars are not shown on the variability plot because conventional and MC‐based results are correlated, instead the difference in variability is shown with the associated error obtained by Equation (8). Error bars for the speedup factor are obtained from Equation (11). MC, Monte Carlo; ROIs, region of interests.

Figure 4 shows the differences in variability for various ROIs in the NEMA phantom patient and the 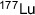
‐DOTA‐TATE patient; in all cases, MC‐based yields statistically significant reduced variability (e.g., variability reduction of (4.19±1.39)% in the 10 mm NEMA sphere). In the NEMA phantom, the largest speedup factor was 2.78±0.33 (32 mm sphere) and the smallest was 1.35±0.22 (13 mm sphere), highlighting significant gains in effective scan time when MC‐based is used for reconstruction. The 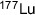
‐DOTA‐TATE patient data consisted of only six noise realizations, so the error bars were large, but a statistical analysis on all five ROIs rejects the null hypothesis that SF≤1 (p=0.0489, one‐sided *z*‐test); this indicates a high probability of MC‐based recon producing reconstructions with reduced variability.

## 4 DISCUSSION

The initial simulated phantom study demonstrated that TEW scatter correction causes positive bias in contrast for hot ROIs in a warm background (116.9% RC for 72 mm sphere). This is reflected in the NEMA phantom experiment, where the RC versus sphere diameter curve exceeds 100% at large radii for conventional reconstruction, but converges close to 100% for hybrid‐MC reconstruction (Figure 3). Uptake in smaller ROIs from conventional reconstruction have (i) lower net bias caused by cancelation of scatter (positive) and resolution (negative) related biases but (ii) higher sum of absolute biases; the sum of absolute biases is a more important metric, particularly, if bias due to TEW depends on scatter conditions, such as the phantom and patient geometry. For example, RCs obtained using conventional reconstruction for a NEMA phantom geometry may be inapplicable to a patient use case since the scatter conditions are different. While the RCs for hybrid‐MC reconstruction may be lower, application of RCs may be more consistent under different scattering conditions since the net bias is primarily due to finite resolution. Future studies should explore imaging of real phantoms with large sphere diameters (e.g., ≥ 70 mm) to confirm the results demonstrated here.

It should be noted that the RC for the 40 mm sphere of the simulated NEMA phantom (92.1%) differed from the RC of the real 37 mm sphere in the NEMA phantom (73.6%) despite them being nearly the same size. There are multiple potential reasons for this: (i) the radial distance in the real phantom is larger on average (18.4 cm) than that of the simulated phantom (15 cm), thus resulting in reduced resolution and subsequently smaller RC, (ii) the SIMIND physics model was not sufficient accurate to model all phenomena in the real imaging scenario, (iii) the ROIs obtained on the CT were misaligned with the SPECT volume. The effect of (i) can be mitigated in future studies via matching phantom sizes and radial paths between simulated and acquired data. The effect of (iii) can be mitigated by using phantoms with cold background and using oversized ROIs to capture activity outside the sphere (this may mitigate partial volume effects and enable study of bias purely due to inadequate scatter correction).

**FIGURE 5:**
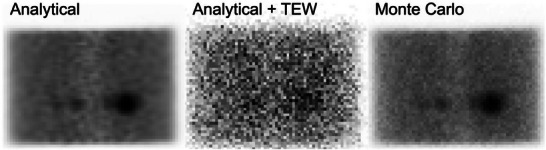
Left: analytical forward projection (without the scatter term included). Middle: analytical forward projection with scatter additive term estimated via TEW. Right: Monte Carlo forward projection. The phantom used as input for forward projection corresponds to the reconstruction of the first NEMA acquisition in Figure 2 at 10 iterations. TEW, triple energy window.

Variability was also reduced in all ROIs in both the NEMA phantom and patients. The effective scan time increased by as much as a factor of 2.78±0.33 (32 mm sphere, NEMA phantom) and 2.04±0.89 (left kidney, patient). This yields a few possibilities: (i) scans can be acquired for the same amount of time and MC can be used to improve precision, (ii) patient scans can be acquired for less time and reconstructed with a hybrid‐MC approach or (iii) new collimator configurations can be used to improve resolution while maintaining the precision levels of conventional reconstruction. The latter is particularly promising, since it will remove biases due to finite resolution. The reduction of variability using MC forward projection may seem counterintuitive, since MC forward projection introduces stochastic sampling of photon paths, which adds noise to image reconstruction; as shown in Figure 5, however, the noise attained from finite photons in MC forward projection is significantly less than the noise from TEW estimates: this is the basis for the reduction of variance in image reconstruction.

It is apparent in Figure 2 that the hybrid‐MC reconstructions have a visual edge artifact outside the FOV of the phantom. This edge corresponds to the detector radial path; no photons can be simulated outside this boundary in SIMIND. The edge artifact may therefore be a Gibbs artifact due to the sharp change in voxel values at the beginning of reconstruction. It may also be due to acquired counts from stray background radiation that lie outside the FOV of the phantom in projection space; excess counts at the boundary of the reconstruction region may be attempting to account for the presence of such detected events, since the initial voxel values of zero outside the detector path remain zero throughout the reconstruction. It is also apparent in Figure 1 that there is a Gibbs edge artifact in the 72 mm sphere for both the conventional and hybrid‐MC reconstructions; future research may seek to compare the magnitude of such artifacts between conventional and hybrid‐MC reconstruction, and the corresponding impact on RC values.

This paper demonstrated bias and variability reduction for the isotope 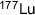, but it is likely to have similar benefits for other isotopes. 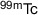, for example, has a higher proportion of scatter counts in its primary window due to lower photon energies with higher probability of scatter. The alpha‐emitting isotopes 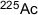 and 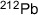 have a significantly low count rate in clinical acquisitions, so modest reductions in variability may significantly improve image quality and quantification. Future research should compare conventional versus MC‐based reconstruction for a variety of isotopes.

The main drawback of MC‐based reconstruction is the substantial increase in computation time. The hybrid‐MC reconstructions were ran on an Azure computer with 96 CPU cores and required approximately 2 h to reconstruct (10 iterations, 8 subsets) versus approximately 30 s for conventional reconstruction (which utilized GPU). It should be noted that consumer‐grade computers do not have 96 CPU cores, so reconstruction times may take an additional five to ten times longer. Practical clinical implementation would likely require access to cloud based computing and the ability to spawn new systems for each set of SPECT data to be reconstructed. GPU‐based implementations and further variance reduction techniques may enable the techniques to be used on consumer‐grade computers.

MC‐based reconstruction has further potential to improve precision by using counts acquired from multiple windows. One approach is to sum counts from multiple windows (e.g., a 113  and 208 keV window for 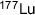) and reconstruct the sum of the projection data. If MC is also included in the back projection operator, the contribution of photons from multiple energy windows (including scatter windows) can be simultaneously reconstructed without summation; this concept can be extended to perform reconstruction on all detected photons, provided their detected energies are recorded (this is effectively using many energy windows). While working on full MC‐reconstruction that uses MC in the back projection, however, has not yet been fully developed and tested, it is postulated that it will lead to substantial improvements of precision for all isotopes.

## 5 CONCLUSION

Hybrid‐MC reconstruction outperformed conventional reconstruction in all quantitative metrics when applied to 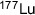 phantom and patient data. Hybrid‐MC reduced scatter‐dependent bias, since TEW inherently overestimates scatter (e.g., RC of 116.9% conventional vs. 101.3% MC‐based in 72 mm sphere of MC simulation data). This negative difference between RC was maintained in ROIs of the real data, indicating that the positive bias incurred from TEW is present in real data. MC‐based reconstruction was able to reduce variability in all ROIs (e.g., by (4.19±1.39)% in 10 mm NEMA sphere), effectively increasing the scan time (e.g., factor of 2.78±0.33 in 32 mm NEMA sphere). MC‐based reconstruction is thus superior to conventional reconstruction in terms of both bias and variability, enabling improved activity distribution accuracy with shorter required acquisition times in 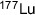 imaging and dosimetry.

## CONFLICT OF INTEREST STATEMENT

The authors declare no conflicts of interest.

## Figures and Tables

**FIGURE A1:**
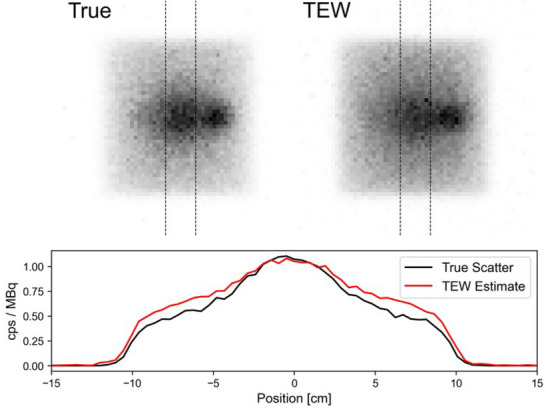
Top: sample projection data from the simulated SIMIND experiment showing true scatter events within the photopeak window and the TEW estimate obtained using adjacent windows. Bottom: vertical profile obtained via summing pixels horizontally between the two dotted lines in the top figure. TEW, triple energy window.

## APPENDIX A

### A.1 Triple energy window bias and noise

Figure A1 shows sample projections from the SIMIND simulated phantom data considered in this paper. Poisson noise was not added; noise in the data corresponds to the finite number of photons simulated in SIMIND. The true scatter events in the photopeak, as well as the estimate obtained using TEW are compared. As can be seen, TEW overestimates the amount of scatter outside the central hot region and slightly underestimates the scatter inside hot region. This consequently leads to a positive bias in contrast after image reconstruction.

Although analytical forward projection is noiseless, the addition of the TEW term adds significant noise to the forward model in MLEM; this is explicitly shown in Figure 5, which shows a forward projection of the reconstructed phantom in Figure 2 (after 10 iterations) using both analytical and MC forward projections. Although some noise is attained in MC forward projection (due to a finite number of 200 million photons simulated per projection), it is significantly less than the analytical forward projection with the TEW estimate added.

### A.2 Uncertainty derivations

The following section contains derivations for Equations (8) and (11). The square of precision $\sigma^{2}$ can be identified as a sample variance. It should be noted that $\sigma_{\mathrm{conv}}^{2}$ and $\sigma_{\mathrm{MC}}^{2}$ are not independent random variables and may have significant correlation since they are ultimately derived from the same set of raw SPECT projection data (albeit via two different reconstruction techniques).

Let $x_{1}\text{…}x_{n}$ represent n observations from a p‐dimensional multivariate normal distribution with covariance matrix $\Sigma ={\left[\Sigma_{jk}\right]}_{j,k=1,\text{…},p}$, and let $\bar{x}$ denote the multivariate sample mean. The sample covariance matrix can be computed as

(A1)
$$\sigma_{jk}=\frac{1}{N-1}\sum_{i=1}^{N}\left(x_{ij}-{\bar{x}}_{j}\right)\left(x_{ik}-{\bar{x}}_{k}\right).$$

Muirhead^27^ (pg 90) showed that

(A2)
$$\mathrm{Cov}\left(\sigma_{jk},\sigma_{lm}\right)=\frac{\Sigma_{jl}\Sigma_{km}+\Sigma_{jm}\Sigma_{kl}}{N-1},$$

implying that the following is a covariance estimator:

(A3)
$$\hat{\mathrm{Cov}}\left(\sigma_{jk},\sigma_{lm}\right)=\frac{\sigma_{jl}\sigma_{km}+\sigma_{jm}\sigma_{kl}}{N-1}.$$

In our case, p=2 (conv and MC) and N is the number of SPECT noise realizations. Equation (A3) can be used to derive the sampling variance of the sample variance (letting $\sigma_{jj}\equiv \sigma_{j}^{2}$)

(A4)
$$\hat{\mathrm{Var}}\left(\sigma_{j}^{2}\right)=\hat{\mathrm{Cov}}\left(\sigma_{jj},\sigma_{jj}\right)=\frac{2\sigma_{j}^{4}}{N-1},$$

as well as the covariance between sampling variances

(A5)
$$\hat{\mathrm{Cov}}\left(\sigma_{j}^{2},\sigma_{l}^{2}\right)=\frac{2\sigma_{jl}^{2}}{N-1}.$$

Uncertainty propagation for the quantity $\sigma_{\mathrm{conv}}^{2}/\sigma_{\mathrm{MC}}^{2}$ (using results from Equations A4 and A5) can be used to derive Equation (11). Uncertainty propagation for the difference in Equation (7), however, requires the quantities $\hat{\mathrm{Var}}\left(\sigma_{j}\right)$ and $\hat{\mathrm{Cov}}\left(\sigma_{j},\sigma_{l}\right)$. These can be approximated via Taylor expansion:

(A6)
$$\hat{\mathrm{Var}}\left(\sigma_{j}\right)\approx \frac{\hat{\mathrm{Var}}\left(\sigma_{j}^{2}\right)}{4\sigma_{j}^{2}},$$

(A7)
$$\hat{\mathrm{Cov}}\left(\sigma_{j},\sigma_{l}\right)\approx \frac{\hat{\mathrm{Cov}}\left(\sigma_{j}^{2},\sigma_{l}^{2}\right)}{4\sigma_{j}\sigma_{l}}.$$

Uncertainty propagation for the quantity $\sigma_{\mathrm{conv}}-\sigma_{\mathrm{MC}}$ (using the results of A6 and A7) can then be used to derive Equation (8). It should be emphasized that this analysis assumed normality assumptions of the underlying data $x_{1}\text{…}x_{n}$, which in our case corresponds to total counts within a volume of interest. Since this may not necessarily be the case, error bars derived in this study are not meant to be exact, but instead provide an approximate order of magnitude.
